# A Mathematical Model towards Understanding the Mechanism of Neuronal Regulation of Wake-NREMS-REMS States

**DOI:** 10.1371/journal.pone.0042059

**Published:** 2012-08-08

**Authors:** Rupesh Kumar, Amitabha Bose, Birendra Nath Mallick

**Affiliations:** 1 School of Life Sciences, Jawaharlal Nehru University, New Delhi, India; 2 School of Physical Sciences, Jawaharlal Nehru University, New Delhi, India; 3 Department of Mathematical Sciences, New Jersey Institute of Technology, Newark, New Jersey, United States of America; 4 School of Computational and Integrative Sciences, Jawaharlal Nehru University, New Delhi, India; Georgia State University, United States of America

## Abstract

In this study we have constructed a mathematical model of a recently proposed functional model known to be responsible for inducing waking, NREMS and REMS. Simulation studies using this model reproduced sleep-wake patterns as reported in normal animals. The model helps to explain neural mechanism(s) that underlie the transitions between wake, NREMS and REMS as well as how both the homeostatic sleep-drive and the circadian rhythm shape the duration of each of these episodes. In particular, this mathematical model demonstrates and confirms that an underlying mechanism for REMS generation is pre-synaptic inhibition from substantia nigra onto the REM-off terminals that project on REM-on neurons, as has been recently proposed. The importance of orexinergic neurons in stabilizing the wake-sleep cycle is demonstrated by showing how even small changes in inputs to or from those neurons can have a large impact on the ensuing dynamics. The results from this model allow us to make predictions of the neural mechanisms of regulation and patho-physiology of REMS.

## Introduction

Sleep and wakefulness have been objectively classified primarily based on electrophysiological signals from the brain, the electroencephalogram (EEG), the antigravity muscles, the electromyogram and eye muscles, and the electrooculogram. Transection, lesion and stimulation studies have shown involvement of specific brain regions in the regulation of wakefulness and sleep (see review in [1]). Extracellular single unit recording and local microinjection of receptor agonist and antagonist in behaving animal models have significantly advanced our understanding of the function of specific neurons, their chemical nature, receptor type and their interactions for such regulation [2], [3]–[5]. Neurons in the rostrally located midbrain reticular formation (MRF) are responsible for waking. Those located caudally (medullary) in the brainstem reticular formation (CRF) and in the preoptic anterior hypothalamus (POAH) are responsible for sleep [1], [6]. Associated studies revealed that the anterior part of the basal forebrain including POAH is responsible for sleep, while the posterior portion for waking [1], [7]. The sleep and waking modulatory sites possess relatively more number of neurons whose firing rates increase during their respective behavioral states. Sleep and wake active neurons are reciprocally connected e.g. neurons in MRF and POAH [8]–[12] and MRF and CRF [13]–[15]. A feed forward excitatory influence from CRF to the POAH hypnogenic area has also been established [10], [16].

The sleep state has been further divided into rapid eye movement sleep (REMS) and non-REMS (NREMS); the former normally appears only after NREMS and not following waking. REMS is regulated by the interactions between REM-on and REM-off neurons, which normally behave in a reciprocal manner; activity of the former increases whereas that of the latter decreases during REMS [17]–[20]. Subsequent studies delineated various neuronal groups, their neurochemical nature and their roles in various functions involved with sleep-waking [21]–[25]. For example, noradrenalin (NA)-ergic neurons are concentrated in locus coeruleus (LC), the site of REM-off neurons, cholinergic neurons in latero-dorsal tegmentum/pedunculo-pontinetegmentum (LDT/PPT), the site of REM-on neurons, serotonergic neurons in raphe, and histaminergic and orexinergic neurons in the postero-lateral part of the hypothalamus. The influence of various neurotransmitters and subtypes of receptors on neurons in LC and LDT/PPT on modulatory effect of REMS has been studied [26], [27]. Further, the roles of pre- and post-synaptic connections in LC [28], [29] and PPT [30], [31] for modulation of release of neurotransmitters and their effects on REMS regulation have also been studied. These findings have been synthesized into a working model of neuronal connections for REMS-regulation that has been proposed recently by Mallick et al. [32]. Validating the proposed functional model of Mallick et al. through mathematical modeling is one of the primary aims of this paper, so that the latter model can be used to extrapolate and predict detailed neural regulation of REMS during normal and REMS-associated patho-physiological conditions.

Sleep-waking and their rhythms are affected by host of neurotransmitters including orexin (ORX). The ORX-ergic neurons are located in the perifornical area and they project to wake promoting, NREMS promoting, as well as to REMS regulating areas [33], [34]. Various neuronal groups that participate in sleep-wake cycling are also subject to inputs from many other regulatory sites including a homeostatic sleep drive and circadian rhythm as proposed in the two-process model [35], [36]. Thus, it is reasonably convincing that regulation and sequential expression of waking-NREMS-REMS is inter-related as well as dependent on multiple, complex factors.

Most *in vivo* studies are usually conducted by manipulating activity of neurons located in one brain area and studying the effects on a behavior, as for example waking-NREMS-REMS in this study. However, neurons in the brain are in a dynamic and interactive state. Experimentally it is difficult to simultaneously manipulate neurons at multiple locations *in vivo* and to carry out repeated studies. Mathematical modeling of known neural circuits based on *in vivo* biological data gives us a reasonable handle to overcome these limitations. Indeed several models have been proposed based on published data that attempt to integrate a number of experimental findings to understand regulation of sleep-waking [37]–[45]. Nonetheless, several questions remain unresolved by prior models. For example, i) how firing of REM-on neurons is initiated for REMS regulation; ii) why REMS does not appear during waking but appears only after a period of NREMS; iii) what is the mechanism for the reduction of firing of REM-off neurons during REMS; iv) what would happen if REM-on neurons were activated during waking; v) what effect would activation of REM-off neurons have during REMS; and vi) how does a neurotransmitter system, such as the ORX system, balance the inputs it receives to promote stability of sleep-wake cycling.

Based on animal data, including a significant proportion from our lab, we present a comprehensive mathematical model for the regulation of waking-NREMS-REMS cycle and use it to probe the mechanisms that may underlie different rhythms and transitions between them. Through simulations and analyses, we show that the model reproduces several of the known aspects of sleep-wake cycling such as rapid transitions between NREMS and wake, the correct phasing of the onset of sleep with respect to the circadian rhythm and prolonged length of sleep recovery after sleep deprivation. We then address the questions raised above. We confirm the recently proposed role of pre-synaptic inhibition on the inhibitory NA-ergic terminals of LC-REM-off neurons acting on cholinergic REM-on neurons in PPT for initiating REMS [32], which is difficult to show experimentally by *in vivo* studies. We use the model to suggest that inhibition from MRF prevents accidental onset of REMS during waking that apparently may be at least one of the causes of narcolepsy. We also investigated the role of ORX-ergic neurons in stabilizing sleep-wake cycling. In particular, we propose that ORX cells act as a gatekeeper to balance the inputs of the circadian rhythm and the feedback from the REMS sub-circuit to force consolidated periods of both wake and sleep. Our proposed model may be used to explore and investigate other questions involving waking-NREMS-REMS regulation in normal as well as in pathological conditions.

## Materials and Methods

### 2.1 Mathematical Model Formulation

Since our modeling study aims to understand the basic architecture and mechanism of transition between different sleep wake states, we modeled the mean population activity of each group of neurons. This approach has been successfully used in other studies [38], [40], [44], where the heuristic activity level was modeled to change between low and high values. We used the Morris-Lecar (ML) equations [46] to model the mean activity of each neuron group.


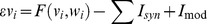
1


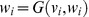
2

Here *v_i_* represents the activity of each population and is scaled to vary between 0 and 1. *w_i_* is the recovery variable. *F* and *G* are nonlinear terms that possess certain geometric features described below (see Appendix S1 for complete set of equations).
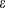
is a small parameter discussed later. *I_syn_* represents the synaptic current from one population to another and is given by


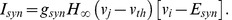
3

Here *g_syn_* represents the strength of the synapse,
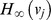
is a Heaviside function that models the effect of the synapse from population *j* and *v_th_* is the synaptic threshold. *E_syn_* is the reversal potential which determines whether the synapse is excitatory or inhibitory. *I_mod_* refers to modulatory input arising due to either the homeostatic drive or the circadian rhythm.

**Figure 1:**
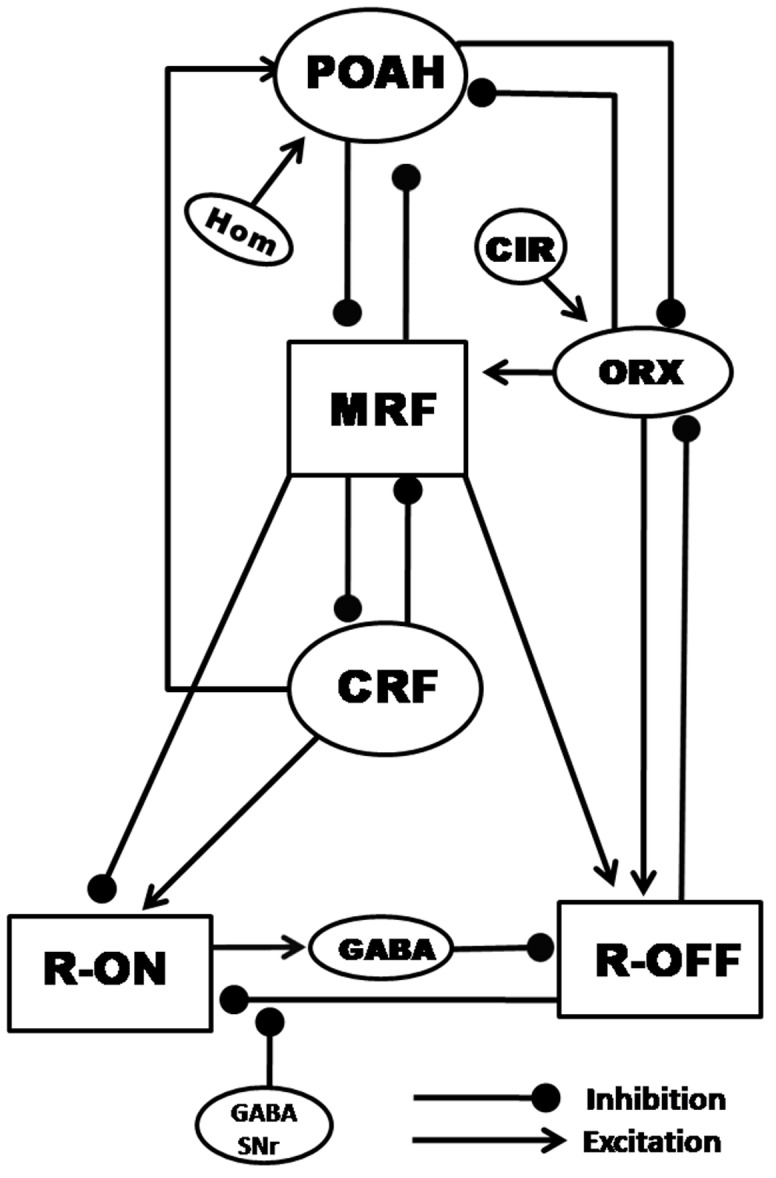
Schematic depiction of the model. POAH- Pre-optic anterior hypothalamus, MRF- Midbrain reticular formation, CRF- Caudal reticular formation, R-ON- REM active neurons (LDT/PPT), R-OFF- REM inactive neurons (LC), GABA-LC- GABA-ergic interneuron in LC, ORX- Orexin-ergic neurons, CIR- Circadian pacemaker from SCN, GABA-SNr - GABA-ergic input from substantia nigra pars reticulate, Hom- Homeostatic sleep drive.

Our model (shown in Fig. 1) includes the sites in the MRF that were found to be most effective in evoking cortical EEG activation upon stimulation [1], [6] as wake promoting. We considered neurons located in the POAH [47]–[50] and neurons in CRF [16], [51]–[53] which showed an immediate and sustained cortical synchronization upon stimulation as the NREMS promoting areas. Several workers have investigated the interaction between the basal forebrain hypnogenic area and the MRF [8], [10], [12], [54], [55]. These studies showed the existence of mutual inhibitory interaction between POAH and MRF [9] forming the basis of a flip-flop circuit as was later proposed [56]. Similarly, the mutual inhibition between the brainstem hypnogenic region (CRF) and midbrain arousal inducing region (MRF) largely comes from the work by Mancia’s group [13]–[15], [57] who showed that, CRF when electrically activated, exerts a short but powerful inhibitory action on MRF neurons and vice-versa. CRF neurons exhibit excitatory effects on POAH neurons [10], [11]. The ORX-ergic system provides a modulatory role by exciting the wake promoting neurons in MRF [58] and its loss results in pathological conditions like narcolepsy [34], [59]. In this study we call the MRF, CRF, POAH and ORX groups of neurons as the WAKE-NREM sub-circuit.

The generation and maintenance of REMS is governed by the shutting off of the NA-ergic REM-off neurons in LC and simultaneous activation of the cholinergic REM-on neurons in LDT/PPT, forming a flip-flop circuit [19], [60] with GABA-ergic interneurons in LC (GABA-LC) between the REM-on to REM-off connection [61]. We allow the GABA-ergic neurons (GABA-SNr) from the substantia nigra pars-reticulate [30], [31] to pre-synaptically modulate the REM-off terminals projecting on the REM-on neurons [32]. This pre-synaptic inhibition prohibits the inhibition from NA-ergic neurons from reaching cholinergic neurons and, as we will show, acts as a trigger for REMS initiation. We call the REM-on, REM-off, GABA-SNr and GABA-LC groups of neurons the REM sub-circuit.

We have incorporated feed-forward input from the WAKE-NREM sub-circuit to the REM sub-circuit in a few different ways. REM-on neurons receive excitatory input from CRF and inhibitory input from the wake promoting area MRF. REM-off neurons receive excitatory inputs from the wake promoting area MRF [62], [63]. REM-off neurons also receive dense excitatory ORX-ergic innervations [64], [65]. Conversely, these neurons exert a negative feedback by hyperpolarizing the ORX-ergic neurons [24], [66]. The inhibition from REM-off to ORX neurons represents the sole feedback from the REM sub-circuit to the WAKE-NREM one.

The role of ORX on sleep inducing area is less well understood. Some reports [25], [58] have shown that injection of ORX in these areas increases wakefulness and decreases both NREMS and REMS. As the sleep promoting neurons in POAH lack the ORX-ergic receptors, ORX-ergic neurons are likely to inhibit POAH neurons by increasing the activity of other arousal related neurons, presumably thereby stabilizing the flip-flop circuit [67], [68]. To simplify this, we included a direct inhibitory effect from ORX-ergic to POAH neurons. POAH also provides a projection back to the lateral hypothalamus, which may inhibit the ORX-ergic neurons [68]–[70].

There are two underlying sources of modulation within the network. The homeostatic sleep drive provides modulation that increases while awake and decreases during sleep [71]. The homeostatic drive to the sleep promoting areas is modeled by


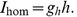
4

The variable *h* is governed by the equation


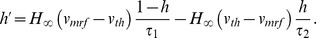
5

This form of homeostatic drive is consistent with the assumptions of the two process model [35], [36]. The variable *h* increases during waking and decreases during sleep. In our model, this homeostatic drive is assumed to target POAH neurons helping them become active to initiate the transition to NREMS. The other form of modulation, on the time scale of the circadian rhythm, is provided by the ORX-ergic system [34], [58]. We allowed circadian input to target ORX-ergic neurons with the following current


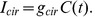
6

The form of *C(t)* was chosen to be consistent with the model by Achermann and Borbely [71], [72] (see Appendix S1 for details). This external current which drives ORX-ergic neurons represents the activity from the circadian pacemaker in the suprachiasmatic nucleus (SCN) [73].

The synaptic inputs that each population receives are modeled using I*_syn_* given above in (3) but now identified with a specific set of synapses. Together with equations (4) and (6) for the modulatory currents, each group receives the following inputs (we use R-off and R-on as abbreviations for REM-off and REM-on both in the equations and figures):

POAH: I_MRF_→_POAH_ + I_CRF→POAH_+ I_ORX_→_POAH_ + I_hom._

MRF: I_POAH_→_MRF_ + I_CRF_→_MRF_ + I_ORX_→_MRF._

CRF: I_MRF_→_CRF._

ORX: I_POAH_→_ORX_ + I_R-off_→_ORX_ + I_cir._

R-off: I_MRF_→_R-off_ + I_ORX_→_R-off_ + I_GABA-LC_→_R-off._

R-on: I_CRF_→_R-on_ + I_MRF_→_R-on_ + I_R-off_→_R-on._

GABA-LC: I_R-on_→_GABA-LC._

**Figure 2:**
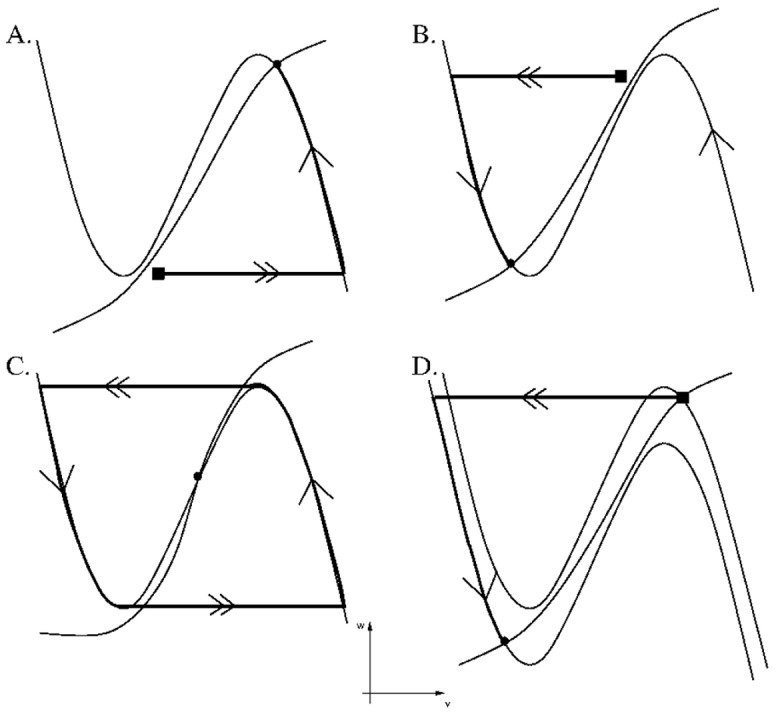
Nullclines and trajectories showing different intrinsic and synaptic behaviors. In panels A, B and D, filled squares denote position of initial conditions that evolve toward particular fixed points (solid circles). Single arrows denote slow flows; double arrows denote fast transitions. A. Nullclines for an intrinsically active cell group intersect on the right branch of the *v*-nullcline. B. Nullclines for an intrinsically silent cell group intersect on the left branch of the *v*-nullcline. C. Nullclines for an intrinsically oscillatory cell group intersect on the middle branch of the *v*-nullcline (open circle). D. Inhibition to an intrinsically active group lowers its nullcline causing the trajectory to move from the solid square location, to the solid circle location.

We next describe the dynamics of the individual population groups and how the above equations determine the level of activity of each group. We first note from equations (1) and (2) that the set of points {(*v,w*): *F(v,w) = 0*} is cubic shaped with left, middle and right branches and is called the *v*-nullcline. The set of points {*(v,w):G(v,w) = 0*} is sigmoidal shaped and is called the *w*-nullcline. In the absence of input, each population group has a base intrinsic level of activity. The MRF group was assumed to be an active population. In the absence of outside input, this was modeled by allowing the *w_mrf_-*nullcline to intersect the right branch of the *v_mrf_*-nullcline to create a stable fixed point at a high value of *v_mrf_* representing high activity (Fig. 2). ORX-ergic and REM-off neurons were also assumed to be intrinsically active. Alternatively, POAH, CRF and GABA-LC were assumed to be silent neuronal groups. This was modeled by making their respective *w-*nullclines intersect along the left branch of their *v-*nullclines at low activity levels. REM-on neurons were assumed to be oscillatory. By this we mean that in the absence of input these neurons would alternate between being active and silent. This was achieved by allowing their *v*-nullclines to intersect along the middle branches of their respective *w*-nullclines.

The small parameter ε mentioned earlier in equation (1) separates slow and fast time scales. In particular, because of it, trajectories were forced to spend a majority of their time in a neighborhood of either the left or right branch of a relevant *v*-nullcline. Their evolution along or near these branches is referred to as a slow flow (single arrows in Fig. 2). Alternatively, there are places in the phase space where the trajectory was forced to make a fast transition (double arrows in Fig. 2) between the left and right branches of a cubic nullcline. During these moments, the *v* variable changed very quickly while the *w* variable remained effectively constant.

## Results

The network architecture that we consider (Fig. 1) essentially consists of two distinct flip-flop circuits, one governing wake/NREMS transitions and the other governing NREMS/REMS transitions. While these two sub-circuits are reciprocally coupled to one another, the dominant direction is the feed-forward input from the WAKE-NREM sub-circuit to the REM sub-circuit. Thus in order to analyze the model, we shall first investigate the WAKE-NREM sub-circuit to understand its dynamics under normal conditions. We then turn to the REM sub-circuit to investigate circumstances that give rise to REMS activity. We will then couple the two sub-circuits to determine the manner in which they interact. This approach will allow us to understand how specific groups, synapse types and modulatory inputs affect the overall output of the network.

In any reciprocally inhibitory circuit, there exists multiple ways to transition from one cell group being active to the other becoming active. Among the most common ways that occurs is that one group either “escapes” or is “released” from inhibition of the other group [74]. In escape, the suppressed or inactive group of neurons reaches an appropriate threshold and then becomes active prior to the removal of inhibition. In this case, the inactive group is in control or is responsible for the switch. In release, the active group must become silent first or at least fall below some relevant threshold, releasing the suppressed group from inhibition; here the active group is in control. In what we present below, escape and release mechanisms still occur, but are modulated by the homeostatic and circadian inputs. In turn, this has implications for how we choose to model particular transitions. In particular, while empirical data sometimes suggests which mechanism is most likely, the model can be used to understand the reasons that underlie various switches from one behavior to another.

### 3.1 Regulation of Sleep Wakefulness: the WAKE-NREM Sub-circuit

**Figure 3:**
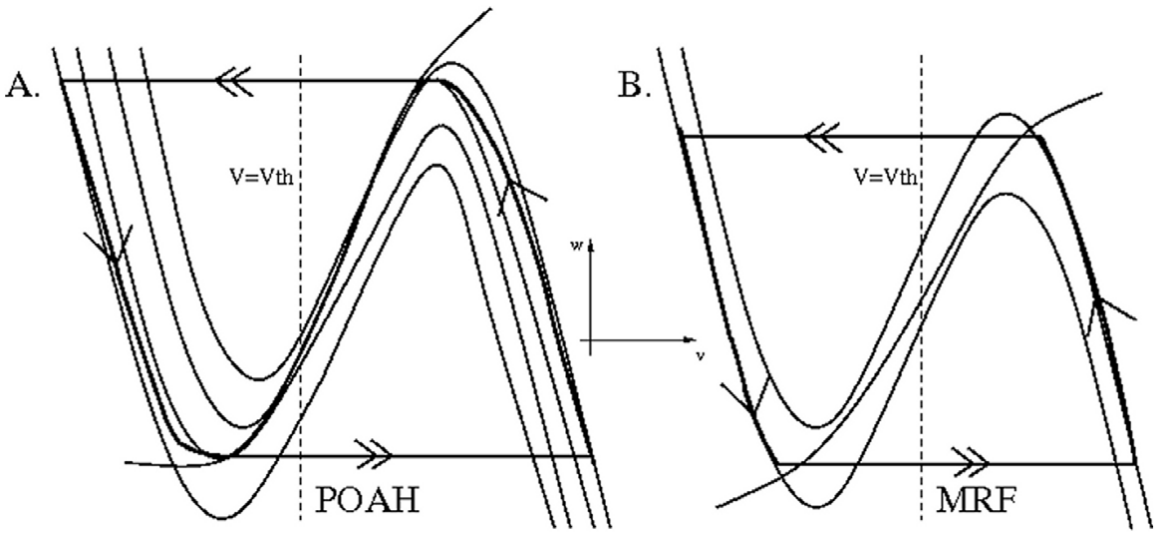
The behavior of POAH and MRF in their respective phase planes corresponding to the voltage traces of Fig. 4. The dashed vertical line corresponds to the synaptic threshold *v_th_*. In panel A. there are four different *v*-nullclines. The lowest one occurred during wake when the inhibition from MRF and ORX was present and the input from the homeostasis was at its smallest. The one above this corresponded to an increase in the homeostasis to just high enough to allow POAH to escape from the MRF and ORX inhibition. The third one corresponded to when POAH reached a local maximum of a *v*-nullcline indicating the end of the sleep state. The highest nullcline corresponded to when the system has just fallen asleep, the homeostasis was at its highest level and there was no inhibition from MRF and ORX. Note that the POAH trajectory was constantly changing the nullcline on which it lies. These nullclines were slowly shifting due to the changes in homeostatic and circadian input, so the POAH trajectory lay on a family of such nullclines bounded between the highest and the lowest.

We first describe how the isolated WAKE-NREM sub-circuit consisting of the POAH, MRF, CRF and ORX-ergic groups, subjected to homeostatic and circadian inputs, governs the basic sleep-wake cycles. Figure 3 shows the behavior of POAH and MRF in their respective phase spaces; CRF was slaved in anti-phase to MRF and has not been shown.

Assume that the system started in the wake state defined by *v_mrf_* > *v_th_*. In this state, ORX-ergic and MRF neurons were active, while POAH and CRF neurons were not. POAH received inhibition from both ORX and MRF as well as input from the homeostatic drive *I_hom_*. The effect of this drive was to slowly increase *v_poah_*, by moving the *v_poah_*-nullcline up in the phase space until the fixed point on the left branch of this nullcline bifurcated; the second lowest nullcline in Fig. 3A. Contributing to the ability of POAH to escape from inhibition was that the inhibition from ORX to POAH was also decreased due to reduced circadian drive to ORX. This allowed the *v_poah_* trajectory to jump on the fast time scale to the active state such that *v_poah_* > *v_th_*. Next, the inhibitory inputs from POAH to MRF and ORX were quickly activated, lowering their *v*-nullclines and forcing their trajectories to transition on the fast time scale to the silent state (Fig. 3B). In particular, *v_mrf_* < *v_th_*, indicates that the system was now in the sleep state. Because of the mutually inhibitory synapses between MRF and CRF, when *v_mrf_* < *v_th_*, this implied that *v_crf_* > *v_th_*. Thus, both POAH and CRF were simultaneously active during sleep states. During the time that the system was in the sleep state, the homeostatic drive to POAH was decreasing, thereby lowering the *v_poah_*-nullcline in phase space. At the same time, the POAH trajectory moved up the right branches of the set of *v_poah_*-nullclines. Similarly the MRF trajectory moved down the left branch of the *v_mrf_* -nullcline towards the local minimum. At this stage, two possibilities existed for how the system transitioned back to the wake state. First *v_poah_* could reach a local maximum along the family of right branches of the *v_poah_*-nullclines. This would signify that the homeostatic sleep drive was too weak to keep the system asleep. Second, *v_mrf_* could reach a local minimum of its nullcline, implying that the wake-active neurons have escaped from suppression. We have chosen parameters so that the inhibited left branch of the *v_mrf_* -nullcline intersects the *w_mrf_* -nullcline. Thus the *v_mrf_* trajectory cannot escape from inhibition, but must be released when the *v_poah_* trajectory reaches a local maximum of the appropriate *v_poah_*-nullcline. In particular, the MRF trajectory made sharper transitions between silent and active states from locations that are not local extrema. Thus, in our model, the transitions between both the sleep to wake state and the wake to sleep state were controlled by POAH activity through the modulation of *v_poah_* by the homeostatic and circadian drive. One specific consequence of this assumption is related to how our model behaves during sleep deprivation and is discussed below.

**Figure 4:**
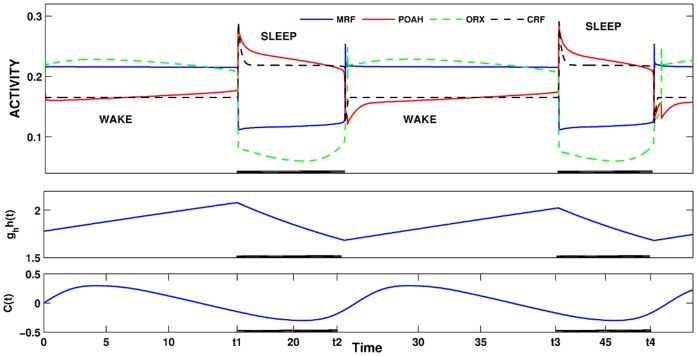
Homeostatic and circadian regulation of sleep-wake cycling. Dark bars indicate the times during which the system was asleep. The wake-promoting neurons MRF were active during wake. During this time, the homeostatic drive increased preparing the system for sleep. When large enough, it allowed the sleep-promoting POAH neurons to become active. Reciprocal inhibitory connections between MRF and POAH and MRF and CRF helped in the transition between states. During sleep, the homeostatic drive decreased, but the length of the sleep cycle was determined by intrinsic properties of POAH as is shown more clearly in the phase plane in Fig. 3. The circadian rhythm modulated the onset of sleep as will be described in section 3.4.

Figure 4 shows the typical activity traces of this sub-circuit and how changes in *I_hom_* induce changes in state. Note the rapid and abrupt onset and offset of *v_mrf_* activity consistent with its trajectory being released from both the active and silent states. The *v_poah_* trajectory also abruptly increased at sleep onset when the homeostatic drive was large enough. Thus, although POAH has escaped MRF and ORX-ergic inhibition, it was only because the homeostatic input allowed it to do so. Alternatively, the homeostatic input played less of a role in determining the transition from sleep to wake. Indeed, under normal model conditions there was no intersection on the right branches of the *v_poah_*-nullclines, meaning that the intrinsic properties of *v_poah_* most strongly determined the length of the sleep state.

Note from Fig. 4 that the homeostatic drive increases when the system is awake and decreases during sleep; however, the circadian rhythm both increases and decreases during these times. This result is consistent with earlier studies where it has been shown that the sleep cycle in rodents typically begins during a decreasing phase of the circadian rhythm and ends on an increasing one [75]. We shall explain why this phase locking occurred in our model in section 3.4 when we discuss in detail the role of ORX.

**Figure 5:**
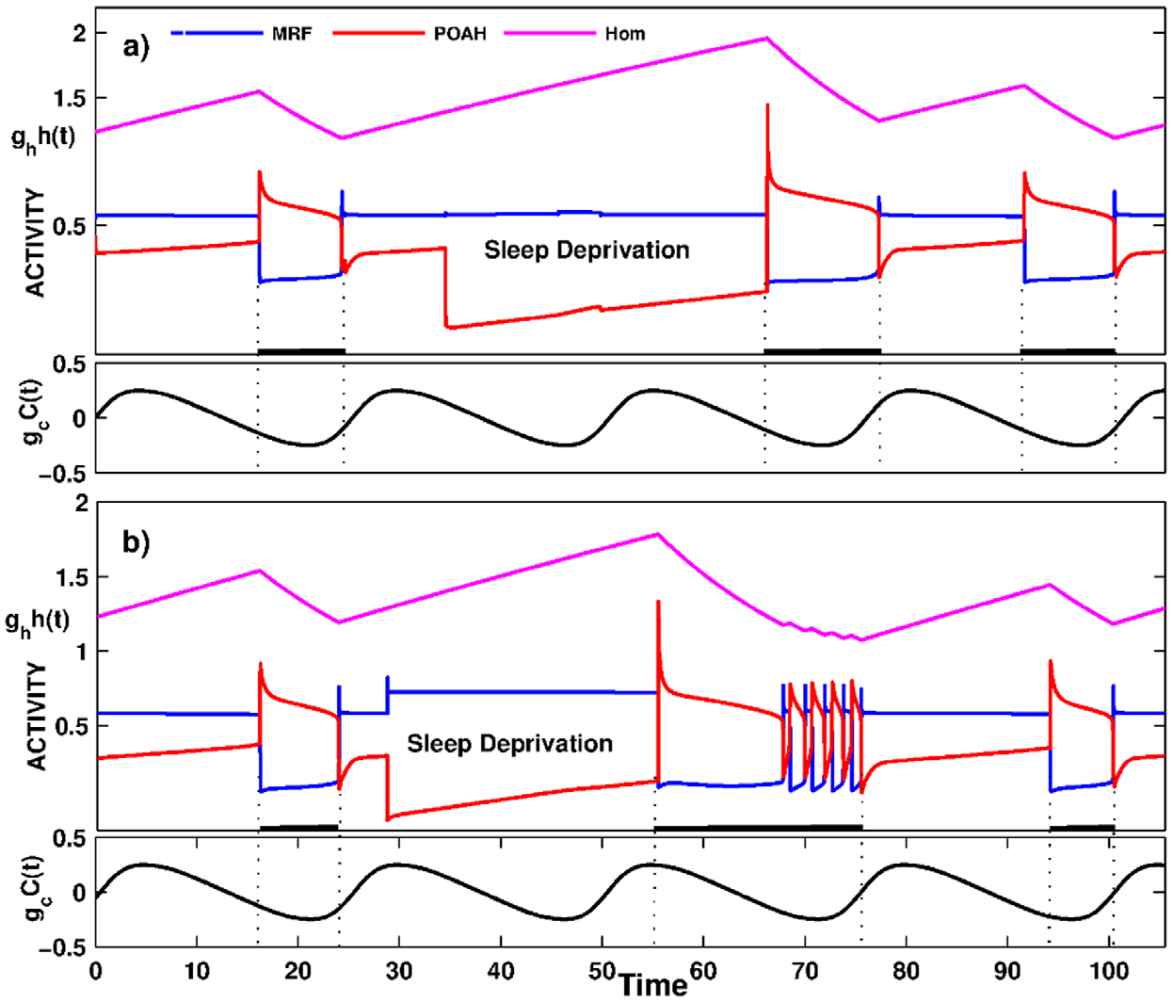
The activity of neuronal groups during a sleep deprivation experiment. The parameters were tuned so that the activity of MRF was high and the activity of POAH was low during the second sleep wake cycle. The subsequent rebound sleep episode was longer in duration than the previous one. Depending on what phase of the circadian rhythm sleep deprivation was interrupted, brief awakenings appeared at the end of a sleep episode. Compare Panels (a) and (b).

The model’s response to sleep deprivation was also consistent with empirical data in rodents [75] and in humans [76], [77] where sleep deprivation, which was associated with increased wake duration, induced increased rebound sleep duration during the recovery period i.e. after the deprivation was stopped. Figure 5a shows one such simulation in which the system was kept awake for substantially longer than normal by transiently suppressing *v_poah_* activity. As can be seen from the trace, the next sleep bout was longer than during normal conditions. This is reasonably easy to explain using phase plane analysis. A longer wake state meant that the homeostatic sleep drive had longer to build up and grew to larger values than normal. Thus, when the system was allowed to fall asleep, the *v_poah_*-nullcline was raised higher in the phase plane than it normally would be due to the larger homeostatic input. This created an intersection of the *w_poah_*-nullcline with the right branch of the *v_poah_*-nullcline. The system now had to wait in the sleep state for the homeostatic drive to decay enough to cause this transient fixed point to disappear to allow the transition to the wake state. This situation is in contrast to the normal duration of the sleep bout which was determined by the intrinsic properties of *v_poah_*, not of the homeostatic drive. Depending on when sleep deprivation is interrupted, the first transition back to the wake state may be followed by several short episodes of sleep-wake bouts (Fig. 5b). The reason these bouts occurred was that the wake state began at a circadian phase that normally corresponds to the sleep state (see also [41]). Thus, the circadian input *C(t)* to ORX is small, and the ensuing inhibitory input to POAH is also not present. In the model, this phasing is such that it keeps the intersection of the *v_poah_* and *w_poah_*-nullclines on the middle branch, thereby allowing *v_poah_* to oscillate between low and high activity. As the bouts continue, the overall level of the homeostatic drive decreased and the circadian input to ORX increased until the intersection point moved to the left branch and put the system into a prolonged state of wake. Note that in both panels a) and b), the entrainment of the onset and offset of sleep to the circadian rhythm took a few cycles to be re-established. Moreover due to the interplay of circadian and homeostatic input to POAH, the second sleep bout after deprivation might end up being slightly longer (a) or shorter (b) than normal.

We note that the sleep deprivation simulation also provided a rationale for why the transition from sleep to wake may be controlled by the intrinsic properties of POAH neurons. As the model now stands, MRF is blocked from becoming active by the creation of a fixed point on the left branch of its *v*-nullcline due to POAH inhibition. If instead, this transition were to be controlled by the ability of MRF to escape from POAH inhibition (i.e. no fixed point were to exist), then during the subsequent sleep episode following deprivation, a different mechanism would need to be present to prohibit MRF neurons from becoming active too soon. Control of sleep offset by POAH is simpler to implement and, as a result, may be more likely to occur.

### 3.2 Regulation of REM Cycling: the REM Sub-circuit

Next consider the REM sub-circuit that consists of the REM-on, GABA-LC, REM-off and GABA-SNr groups. This network displayed two distinct stable activity patterns depending on the choice of parameters. In one pattern, REM-off neurons were active while both REM-on and GABA-LC neurons were silent. When *v_R-off_* > *v_th_*_,_ the inhibitory input to REM-on neurons forced *v_R-on_* < *v_th_*. The inhibition from REM-off neurons created a stable fixed point on the left branch of the *v_R-on_*-nullcline. In turn, GABA-LC neurons received no input from REM-on neurons and remained silent. In this state, the pre-synaptic inhibition of the synapse from REM-off to REM-on was absent, and REM-off neurons were in control of the sub-circuit.

**Figure 6:**
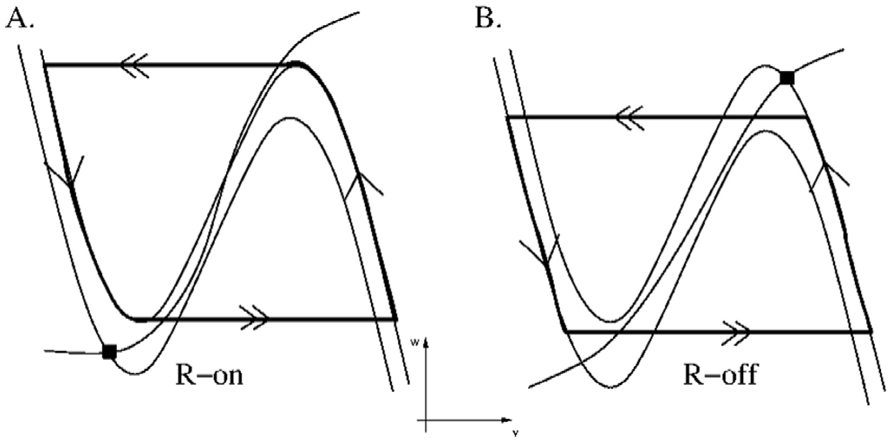
Nullclines and trajectories during REMS and NREMS. Panel A shows REM-on activity while Panel B shows REM-off activity. Solid squares show that the trajectories in the (NREMS) REM-off controlled mode were stuck at fixed points. Trajectories with arrows depicted the oscillatory dynamics in the (REMS) REM-on controlled mode.

The control of the dynamics can be shifted to the REM-on neurons by allowing pre-synaptic inhibition to be present. This functionally destroys the REM-off and REM-on flip-flop making it unidirectional. A second stable solution then arose where REM-on neurons oscillated between silent and active states. This was because the REM-on neurons, in the absence of input, are assumed to be oscillatory, with a fixed point on the middle branch of the *v_R-on_*-nullcline. Their excitatory synapses drove GABA-LC interneurons entraining them to the oscillatory pattern. In turn, GABA-LC interneurons provided rhythmic inhibition to REM-off neurons. The REM-off neurons thus also oscillate between active and silent states. Figure 6 shows how the *v_R-on_* and *v_R-off_* trajectories behave in their respective phase planes. The solid dots represent the position of the trajectories in the REM-off controlled mode, while the trajectories with arrows correspond to the REM-on controlled case. Note that the REM-on trajectory in this mode followed its intrinsic dynamics, while the REM-off trajectory was released or captured by inhibition signified by its trajectory transitioning from points that are not local extrema (much the same way that MRF transitions between wake and sleep as shown in Fig. 3B). Thus the REM-on neurons are in control of the sub-circuit in this state.

**Figure 7:**
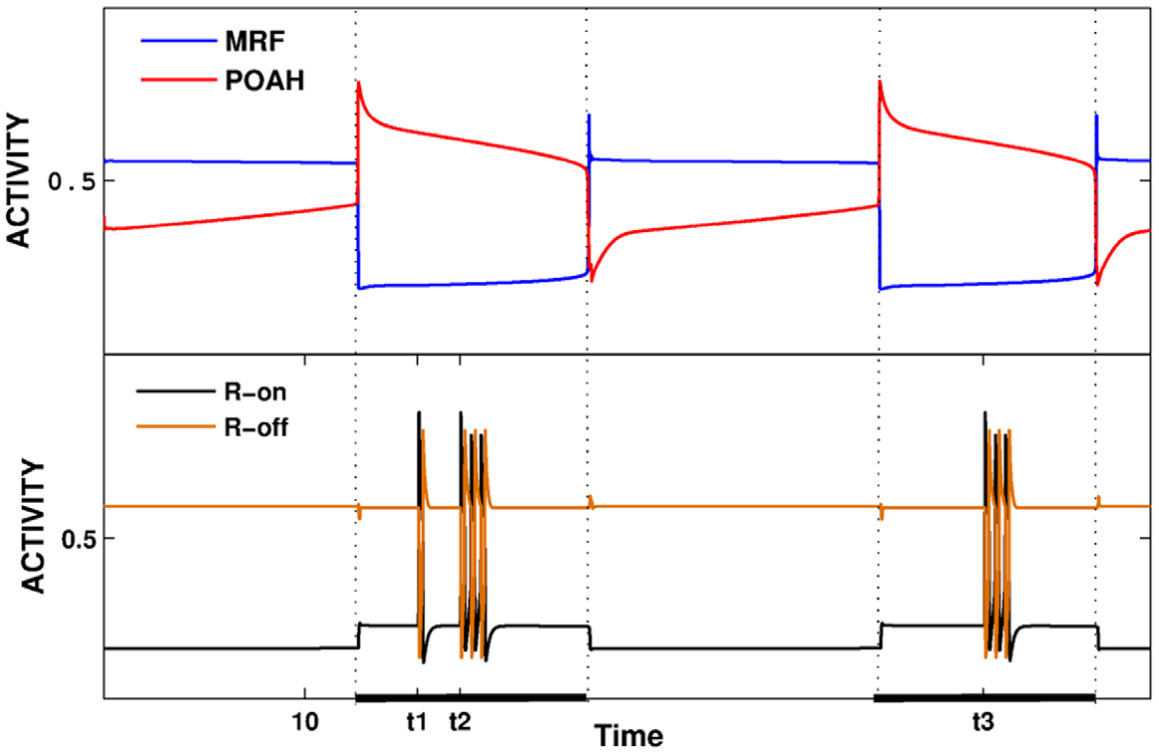
The activity of various neuron groups during sleep-wake cycling. In the first sleep episode, the pre-synaptic inhibition from GABA-SNr was activated at two distinct times, each for different lengths. Pre-synaptic inhibition was triggered once during the second sleep episode. In each episode, this resulted in transient REM-on and REM-off oscillations. The overall transitions between wake and sleep were still governed by the homeostatic and circadian inputs as in Fig. 4.

There is a third activity state that was relevant to the onset of REM-on activity. This was a transient state that was instigated by the transient initiation of the pre-synaptic inhibition of the inhibitory REM-off to REM-on synapse. Brief activation of the pre-synaptic inhibition allowed REM-on neurons to fire transiently, resulting in activation of GABA-ergic interneurons in LC causing suppression of REM-off neurons and initiation of REMS for the duration of the pre-synaptic inhibition. Removal of the pre-synaptic inhibition returns the sub-network to the stable state in which REM-off neurons are active and suppress REM-on neurons. In Fig. 7, we briefly activated the pre-synaptic inhibition from GABA-SNr (twice during the first sleep episode at *t1* and *t2*, once during the second at *t3*). This released REM-on neurons from inhibition allowing the REM bouts to occur. REM-off neurons also oscillate during REM bouts due to the inhibition that they receive via GABA-LC.

REM-on and REM-off neurons can be made to be simultaneously active by blocking the synaptic connection between GABA-LC and REM-off neurons while the pre-synaptic inhibition from GABA-SNr was present (simulations not shown). The pre-synaptic inhibition allowed REM-on neurons to be active allowing these neurons to excite GABA-LC neurons. By blocking the inhibition from these neurons, REM-off neurons were free to remain active. In effect, both directions of the REM-on and REM-off reciprocal inhibitory circuit were disabled in this state and those neurons now followed their intrinsic dynamics.

### 3.3 Feed-forward Input to the REM Sub-circuit

Having understood how the individual sub-circuits control sleep-wake cycles and REMS transitions, now we describe how they work in tandem. Note that the WAKE-NREM sub-circuit sends feed-forward input to the REM sub-circuit. Thus the primary issue is to understand how the input from the former moderates the dynamical output of the latter. There are three sets of feed-forward synapses onto the REMS regulating neurons. One set comes from MRF which provides inhibitory input to REM-on and excitatory input to REM-off neurons. The second set is from CRF which provides excitatory input to REM-on neurons. The third excitatory feed-forward input is from ORX-ergic to REM-off neurons.

**Figure 8:**
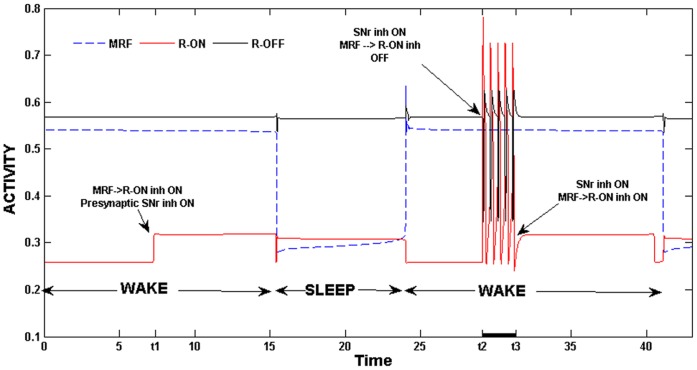
The role of MRF inhibition. MRF inhibition was necessary to prevent REM-on activity during wake. At both the times *t1* and *t2* during wake, pre-synaptic inhibition from GABA-SNr was initiated. This resulted in REM-on activity only in the second case since MRF inhibition was not present. The re-application of MRF inhibition terminated REM-on activity at time *t3.*


Activation of MRF neurons results in slightly raising the firing rate of REM-off neurons, since MRF has an excitatory input to REM-off neurons. This is because excitation causes the *v_R-off_*-nullclines to rise in phase space, pushing the fixed point to occur at higher values of *v_R-on_*. This is consistent with earlier reports [78] where REM-off activity has been shown to be higher during wake than sleep [20]. Removal of this synaptic input, however, does not qualitatively change the output of the model. Next consider the inhibitory synaptic input from MRF to REM-on neurons. During wake, when MRF is active, this inhibition helps to suppress REM-on activity. It acts as a second source of inhibition to REM-on neurons, complementing, and, at times, substituting for the inhibition directly from REM-off neurons. Indeed, during wake, if the pre-synaptic inhibition from GABA-SNr was accidentally triggered, then inhibition from MRF was strong enough to prevent REM-on neurons from firing. In Fig. 8, we show activity traces of the MRF, REM-on and REM-off neuronal groups. At the time labeled *t1* during the wake state, we instigated the pre-synaptic inhibition from GABA-SNr to REM-on. Note that the activity level of the REM-on neurons increases, but the group as a whole does not begin firing. This is because the inhibition from MRF is present and is strong enough to continue to suppress REM-on activity. At the time *t2*, during the second wake episode, we repeated this procedure but also removed the MRF inhibition to REM-on neurons. As can be seen in the figure, REM-on neurons begin to oscillate despite this being the wake state. Reapplication of this MRF inhibition at the time *t3* terminated the REM-on activity. The results shown in Fig. 8 demonstrate that one important role for the MRF to REM-on synapse is to maintain suppression of the latter during the wake state. A second source of feed-forward input to the REM sub-circuit is from CRF neurons that provide excitatory input to REM-on neurons. It has been suggested that there may exist a REMS homeostatic drive that builds up during sleep and helps to control the duration of REMS bouts [38], [79], [80]. The effect of this putative homeostatic drive can be modeled through the CRF to REM-on synapse by letting the strength of this synapse depend on the length of the current sleep bout consistent with the hypothesis of Bennington and Heller [80]. In particular, in equation (3), we replaced the Heaviside function for the CRF to REM-on synapse with a dynamic variable *s* satisfying

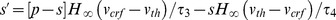



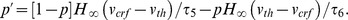


The REMS homeostasis was governed by the equation for the variable *p*, increasing when the system was asleep and decaying while it was awake. The influence of the REMS homeostasis was incorporated into the CRF synapse whenever the system was asleep by making *s* approach *p* with rate 1/τ_3_. We took τ_3_ to be small so that effectively *s = p* during the sleep episode. We also took τ_4_ to be small so that the CRF synapse decayed quickly during wake.

**Figure 9:**
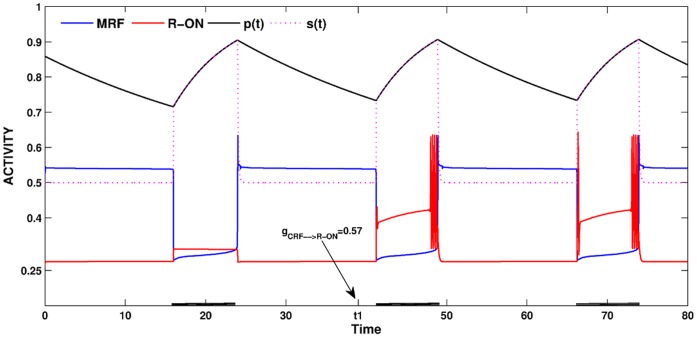
The putative REM homeostat. The strength of the CRF to REM-on excitatory synapse was allowed to depend on the length of the sleep and wake states. At the time denoted *t1*, the strength
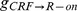
is increased from 0 to 0.57, resulting in REM bouts in subsequent sleep cycles.

At the onset of a sleep bout, the *s* value was very small and the *w_R-on_*-nullcline intersected the *v_R-on_*-nullcline on its left branch. As the length of the sleep bout increased, *s* built up causing the *v_R-on_*-nullcline to move slowly up in phase space. Eventually, *s* built up enough to allow the fixed point on the left branch to bifurcate, thereby instigating REM-on activity. In the current version of our model, once REM-on activity started through this homeostatic mechanism, it continued to occur until the end of the sleep-bout. Figure 9 shows an example where at the time labeled *t1*, we have changed the CRF to REM-on excitatory synapse from 0 to the value 0.57. It was found that REM-on activity began in the middle of the sleep bout as a result of this homeostatic synaptic input. It ended when the system returned to the wake state when the inhibition from MRF to REM-on suppressed REM activity.

The last feed-forward synaptic input from ORX to REM-off neurons, in our model, does not appear to play much of a role in shaping REMS/NREMS dynamics. Rather, it has a similar role to the MRF to REM-off synapses and its exclusion does not qualitatively change the dynamics of the model. This may reflect the fact that ORX-ergic neurons project both to areas possessing REM-on and REM-off neurons [34], [80].

### 3.4 Investigating the Role of Orexin

Loss of ORX-ergic neurons has been demonstrated to play a role in destabilization of the sleep-wake cycle [81]–[83]. ORX-ergic neurons control the sleep-wake switch by exciting arousal inducing neurons in MRF and also indirectly inhibiting sleep related neurons in POAH. In our model, ORX-ergic neurons played a complex role in regulating the dynamics of various neuronal groups in modulating the sleep-wake cycle and this was manifested in at least three ways. First, ORX-ergic neurons transmitted information about the circadian rhythm to the wake-NREM sub-circuit. Second, ORX-ergic neurons were reciprocally coupled via inhibition to POAH neurons which meant that the activity of ORX was indirectly affected by the homeostatic drive. The reciprocal inhibitory inputs to POAH neurons also implied that the ORX-ergic neurons had the potential to indirectly counteract or amplify the effect of the homeostatic drive to POAH neurons. As we show below, this led to behavioral expressions analogous with brief awakenings, comparable to narcolepsy. Third, ORX-ergic neurons were the target for feedback inhibition from the REM sub-circuit via the inhibitory synapses from REM-off neurons.

The circadian modulation passes through ORX-ergic neurons via excitatory synapses to wake promoting MRF neurons [84] and via indirect inhibitory pathways to sleep promoting POAH neurons [67]. Since the model parameters were chosen so that the primary control of switching between sleep and wake lies with the POAH neurons, the excitatory synapses from ORX to MRF played little role. In fact, removing this synapse has no effect on the model dynamics. The effect of the circadian rhythm was primarily transmitted by ORX to POAH. In particular, the inhibition from ORX-ergic neurons, combined with that from MRF neurons, must be overcome by the homeostatic drive in order for the system to fall asleep. Under normal model parameters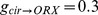, the wake state took up around 65% of the 24 hour circadian cycle, while sleep occupied the remaining 35%. By setting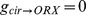, the ratio of wake to sleep was skewed to 71% to 29%, while the overall length of the sleep-wake cycle increased by seven hours. Therefore, the presence of the circadian rhythm increased the percentage of time that the system was in the sleep state. To understand the possible mechanism underlying this, note that without the circadian input, POAH must wait for the homeostatic drive to build up to a higher value to overcome the inhibition of both MRF and ORX. However, when the circadian rhythm was present, ORX activity began to decrease on the downward phase of the circadian rhythm. In turn, this reduced the inhibition from ORX to POAH allowing the latter to escape to its active state with less homeostatic drive and thus earlier than it would have in the absence of the circadian rhythm. As a result, the wake duration was shorter with the presence of circadian rhythm. This result also explains why sleep begins on the downward (waning) phase of the circadian rhythm since ORX to POAH inhibition also decreases in this phase. Note, however, that the increase in the wake to sleep ratio contradicted empirical data of Edgar et al. [85] that suggest that the ratio should decrease with the loss of circadian input. We will later discuss how to bring our model results more in line with the experimental ones.

**Figure 10:**
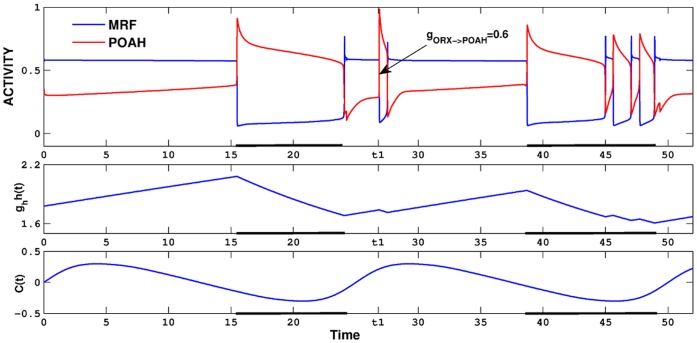
Brief awakenings induced by reducing ORX inhibition of POAH. At the time labeled *t1*, the inhibition from ORX to POAH was reduced resulting in a brief excursion to sleep. The duration of the subsequent sleep cycle was longer, but was interrupted by several brief awakenings. Note the level of the homeostat at the onset of the second sleep cycle was smaller than at the onset of the first due to reduced ORX inhibition.

Keeping the circadian input to ORX at the normal value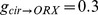, but changing the value of other inputs to or from ORX caused changes to the dynamics within the sleep-wake cycle, but retained the overall 24 hour cycle period. For example, decreasing the amount of inhibition from ORX to POAH caused the system to undergo brief awakenings (see Fig. 10). In the first portion of the figure, we set 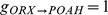 which we then reduced, at *t1*, to 0.6. There were two ensuing effects. First, the system now exhibited brief awakenings toward the latter part of the sleep episode before returning to the fully awake state. Second, the ratio of wake to sleep was skewed to 59% to 41%. These changes in behavior resulted from how the circadian and homeostatic inputs worked together to determining transitions. Since the ORX inhibition to POAH was not present during sleep, it would seem that changes in 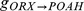 should have no effect on the dynamics. However, the reduction in this parameter did have an effect during wake, with less inhibition implying that the homeostatic drive did not need to build up as much to put the system to sleep; thus the length of the wake state was shortened. Moreover, the lowered homeostatic drive at sleep onset led to changes in the value of the homeostasis when the POAH trajectory reached a local maximum of a *v*-nullcline (this duration was mostly due to POAH intrinsic properties). Additionally, the circadian phase at the onset of sleep was now slightly earlier than normal, leading to a change in the level of ORX inhibition to POAH. As a result, when the POAH trajectory returned to the silent state, evolved down the left branches and was ready to return to the active state, the circadian input to ORX was too low. The ensuing balance of homeostatic and ORX inputs to POAH was then such that there was no fixed point on the relevant left branch of the *v*-nullclines. Thus there was nothing to prevent the system from falling back to sleep. This type of dynamics continued for a few cycles as the overall level of the homeostatic drive continued to decrease slowly. When it eventually reached a low enough level, a fixed point was created on the left branch of the POAH *v*-nullcline and the system switched to prolonged wakefulness.

**Figure 11:**
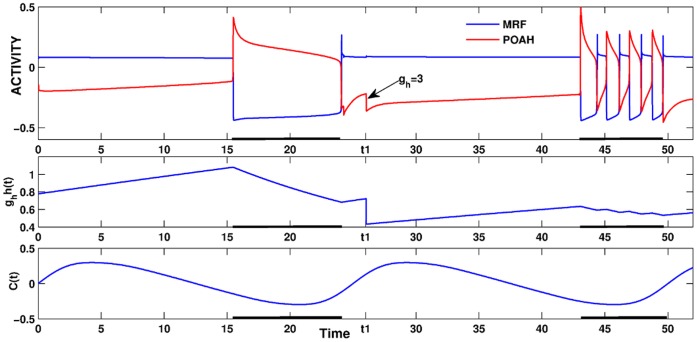
Brief awakenings induced by reducing the homeostatic sleep drive to POAH. At the time labeled *t1*, the maximal strength of the homeostatic drive to POAH was reduced resulting in longer subsequent wake duration and brief awakenings throughout the next sleep bout.

There are other ways to achieve brief awakenings that work on the same principles as changing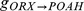. For example, decreasing the strength of the homeostatic drive from 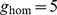 to 3 induced brief awakenings (Fig. 11). In this condition, the ratio of wake to sleep was skewed in the opposite direction to 70% to 30%. Less homeostatic drive in this case meant that the timing of the effect of the circadian rhythm was altered. Namely, when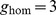, the system had to wait for more of the ORX to POAH inhibition to wear off before it could transition to the sleep state. Thus, the onset of sleep was shifted towards later in the circadian phase (more towards the trough of the rhythm). This had the effect of lengthening the wake state and shortening the sleep state. Additionally, the system went through continual brief awakenings due to the decreased homeostatic drive as described above. Note also that the length of the first sleep bout is much shorter than under normal conditions. This was because the POAH trajectory now reached the active state at a location in phase space that was closer to the local maximum of a right branch of a *v*-nullcline.

**Figure 12:**
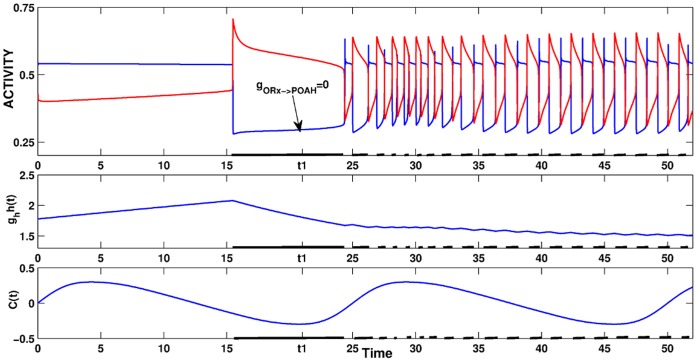
Narcolepsy induced by removal of ORX inhibition. At time *t1*while the system was still asleep, the inhibition from ORX to POAH was removed. At the end of that sleep bout, the system was no longer able to consolidate wake and sleep into distinct episodes. The POAH trajectory was not sufficiently suppressed by the MRF inhibition alone to create a prolonged wake state.

We observed that the total loss of ORX input to POAH completely disrupted sleep-wake cycling. In Fig. 12, 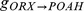
was set to 0 in the middle of a sleep cycle. The system went into an oscillatory state in which sleep and wake alternated, but on a much faster time scale than that of the circadian rhythm. The findings from this simulation were consistent with studies involving narcolepsy [81], [86] which showed that loss of ORX-ergic neurons leads to disruptions in the normal sleep-wake cycle. The reason the system was no longer able to produce consolidated periods of wake and sleep was because the loss of ORX inhibition created an imbalance in the inputs to POAH. Now the MRF inhibition alone was not sufficient to create a fixed point on the left branch of the *v_poah_-*nullcline. Thus the POAH trajectory was free to return to the active state putting the system to sleep. Because of the flip-flop dynamics between POAH and MRF, this in turn caused MRF to oscillate on the same fast time scale, which subsequently prevented the homeostatic drive from building up or decaying properly. The loss of ORX-ergic to POAH inhibition can partially be compensated for by increasing the MRF to POAH inhibition, increasing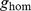, the strength of the homeostatic drive to POAH and weakening the POAH to ORX inhibition (simulation not shown).

**Figure 13:**
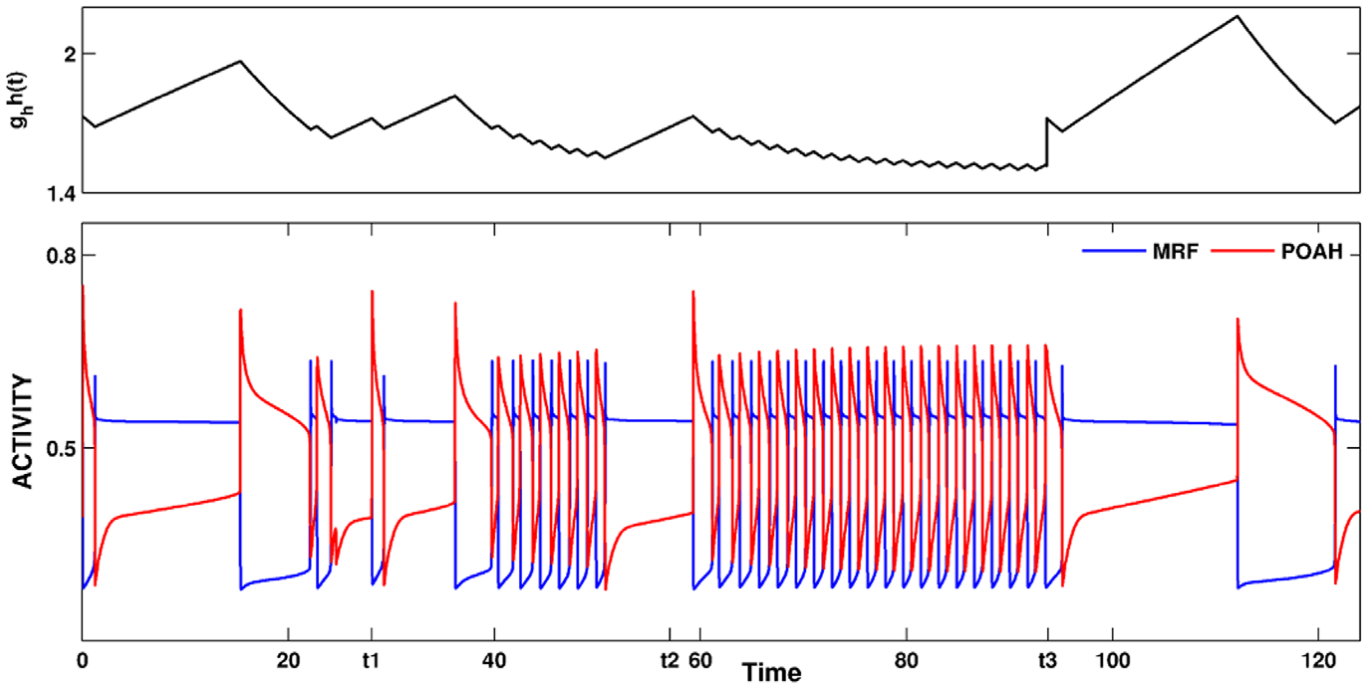
The effect of feedback inhibition to ORX cells from REM-off neurons. The simulation begins with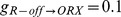. This amount of feedback inhibition did not qualitatively change the basic sleep-wake cycling (compare with Fig. 3). At time *t1*, the feedback inhibition was changed to 0.5. The system was no longer able to stay in a prolonged state of sleep; sleep bouts were continually disrupted by brief awakenings. At time *t2,
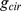
*was set to 0, which destroyed the cycling. At time *t3
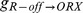
*was reset to 0.1 while *g_h_* was increased to 7 which restored the normal cycling.

The last synaptic connection that we explored was the inhibitory feedback from REM-off neurons to ORX-ergic neurons. This is a particularly interesting synaptic connection as it represents the sole feedback in our model from the REM sub-circuit to the WAKE-NREM sub-circuit. In all prior simulations, we have set the value
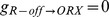
so that there was no feedback. As seen above, one of the primary roles of ORX-ergic neurons was to stabilize sleep-wake cycling. So the primary question to address was what role, if any, this feedback synapse has in either stabilizing or destabilizing sleep-wake cycling. An alternate question to ask was how ORX-ergic neurons balance the feedback inhibition with other inputs they receive. In Fig. 13, the simulation began with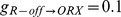. As can be seen in the early part of the trace, this amount of feedback inhibition did not qualitatively change the basic sleep-wake cycling. At time *t1*, we increased the strength of the feedback inhibition to 0.5 and while cycling still occurred, the system was no longer able to stay in a prolonged state of sleep. Instead, sleep bouts were continually disrupted by brief awakenings. Although, there was still consolidation into periods of wake and not being awake, the proper dynamics of sleep were lost. All slow cycling can be destroyed, as is shown from time *t2* onward, by removing the circadian input to ORX cells (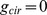). Under such condition there was continual disruption of wake by sleep and vice versa with no consolidated periods of either. This disruption of slow cycling in the absence of circadian input persisted for lower values of 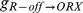
as well. The proper wake-sleep dynamics could be returned even when *g_cir_* = 0 by further reducing
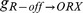
to 0.1 while simultaneously increasing *g_h_* to 7, as was done at *t3*. Thus, this simulation revealed another role of ORX neurons. They must carefully balance the inhibition from the REM sub-circuit to prohibit the feedback from disrupting sleep-wake cycling. Indeed it is an appropriate balance that was missing in the earlier result where the elimination of the circadian input to ORX-ergic cells increased the ratio of wake to sleep in contradiction to the Edgar et al. result [85]. Now with the appropriate level of inhibitory feedback from REM-off to ORX-ergic neurons, the loss of circadian input need not increase the wake to sleep ratio.

## Discussion

Despite being investigated over many decades, the precise neuronal mechanisms governing the waking-NREM-REMS cycle and the regulation of REMS are still not completely understood. While many questions on the role of various neuronal groups have been apparently answered, several are still outstanding. A primary difficulty in addressing many of the questions revolves around the difficulty in experimentally assessing simultaneous contributions of specific groups of neurons located in different anatomical locations to understand a specific behavior, waking-NREMS-REMS in particular for this study. For example, very few experiments *in vivo* have quantified the effect on sleep-waking and REMS in particular by simultaneously manipulating more than one brain area [31], [61], [87]. Most studies involve manipulation of a single brain area under the assumption that all other brain areas continue behaving unaltered. However, this may be unlikely given that several areas in the brain may directly or indirectly dynamically influence other brain areas. Additionally there are other difficulties encountered with *in vivo* experiments in both rodents and humans that limit the ability of experiments to provide definitive answers. Yet this provides a perfect opportunity for exploration using a mathematical model and simulation studies. For example, rodents and humans sleep-wake patterns have certain commonalities e.g. subject to homeostatic and circadian drives and certain differences e.g. polyphasic vs. monophasic. Ideally data obtained and mechanisms discerned from studying one species should provide insight into the behavior of another. Mathematical modeling yields the ability to extrapolate findings between species for exactly this purpose. Namely one can ask the question of whether or not a mechanism say for transitions between NREMS and REMS, found in rodents, is plausible in humans and so on.

In this paper, we have constructed a mathematical model for neuronal interaction that explains various features of the regulation of waking-NREMS-REMS behavior. Our proposed model, based on data collected from rodents and cats and extrapolated to humans, reproduces most aspects of the known neurophysiological behavior of waking-NREMS-REMS. For example, transitions of the mean neuronal activities of the sleep-promoting neurons in the POAH and the wake-promoting neurons in the MRF to and from their respective active states, as observed by the results of this study, are consistent with the *in vivo* findings reported earlier [88], [89]. The system falls asleep on the decreasing phase of the circadian rhythm and wakes up on the increasing phase which is consistent with *in vivo* findings [75]. The model’s response to sleep deprivation by prolonging the wake duration which resulted in increased rebound duration of the subsequent sleep bout is consistent with animal studies [90], [91].

The mathematical model presented in this study has been constructed using the functional model based on *in vivo* animal data that we have proposed recently [32]. Several new insights may be suggested by this study, which apparently might have been difficult to comprehend easily from an *in vivo* behavioral study alone. For example, we are yet to understand how the LC-REM-off neurons become silent during REMS. It was known that REM-off neurons cease firing and REM-on neurons increase firing during REMS. Reciprocal connections between REM-off and REM-on neurons combined with self-inhibitory collateral inputs on the REM-off neurons was proposed to be responsible for rhythmic firing of the REM-off neurons [19], [92]; however, it did not explain complete silence of the REM-off neurons during REMS. Based on a series of *in vivo* studies, we proposed that pre-synaptic inhibition of the REM-off terminals onto REM-on neurons is the likely cause of initiation of REM-on activity, which in turn would inhibit the REM-off neurons [30]–[32]. However, it is extremely difficult to experimentally show the same *in vivo* in behaving animals. Our mathematical model, as constructed in this study, helped to circumvent this difficulty. The results clarified and explained a long standing problem of initiation of REMS by pre-synaptic inhibition through release of inhibitory NA at the REM-off NA-ergic terminals on the REM-on neurons. The said withdrawal of inhibition from the REM-on neurons triggered GABA-ergic neurons, which in turn inhibit the REM-off neurons generating REMS [61]. This pre-synaptic inhibition may arise from the GABA-SNr [31], a component of limbic system and is involved in learning and memory. This knowledge led us to propose that because of activation of those areas, dreams probably appear in conjunction with REMS [32]. The model provides a mechanistic explanation for appearance of dreams during REMS and confirms our recently proposed model based on *in vivo* findings.

We showed the necessity of inhibitory inputs from the MRF to REM-on neurons in preventing the appearance of REMS-like symptoms during wakefulness. In our simulations it was found that if those synaptic inputs were absent then the model predicted that REM-on activity could be instigated during the wake state as well, which may be comparable to hypnagogic hallucinations, dreams that intrude during wakefulness, in psychiatric patients and in narcoleptics [93]. Thus, our findings provide testable hypothesis *in vivo*, into the cellular level causes and mechanisms of this disorder. For example, in principle, one might now attempt to withdraw waking area inhibition from the REM-on neurons *in vivo* (after overcoming the technical limitations though). Similarly, the results from this model may be used to predict testable hypothesis on the presence and/or absence of neuronal connections associated to patho-physiological states as well as in different species in evolution which do not show classical characteristic signs for identifying REMS, which otherwise is extremely difficult to predict and study. For example, in principle, one might investigate if there is any anatomical and functional loss of MRF to REM-on connections in depressed and psychiatric patients, who complain about hallucinations and day dreaming. We understand that considerable technical limitations need to be overcome prior to conducting these studies *in vivo*.

We focused our attention on understanding how the homeostatic- and circadian-drives interact to determine wake-NREMS-REMS rhythms. In particular, we investigated the role of ORX-ergic neurons which are also the targets of circadian drive [73]. These neurons play a very important and delicate role in balancing excitatory and inhibitory inputs on sleep-promoting POAH neurons. Even a small modulation of ORX-ergic neuronal activity or strength of synaptic input on them can lead to large scale changes in sleep-waking. For example, weakening of the ORX-ergic inhibition on to the POAH neurons is at least one of the prominent causes of brief awakenings. The results of our model are consistent with the experimental *in vivo* studies [81], [82], [94] which showed that loss of ORX-ergic neurons was accompanied by multiple transitions across the state while the total duration of states remains unaltered. Complete loss of ORX-ergic inhibitory input to POAH completely disrupting normal sleep-wake cycling is also consistent with the findings of earlier studies [34].

There have been several prior mathematical modeling studies that have addressed some aspects of sleep-wake cycling. Diniz Behn et al. [38] derived a model based on the mouse basic sleep-wake cycle which accounts for transitions between states as well as expressions of brief awakenings. Although the model also included a generalized sleep homeostatic drive and also proposed a homeostasis for REMS, the model principally focused on brief awakenings within sleep. In a follow up study [40], Diniz Behn et al. considered the role of ORX-ergic inputs in stabilizing the sleep-wake cycle. They showed that loss of ORX-ergic activity led to fragmented cycling similar to what was observed in narcolepsy patients. However, neither of these two studies included an explicit circadian drive, nor did they explain the mechanism of silencing of the REM-off neurons for the regulation of REMS. It may be emphasized that in the absence of cessation of REM-off neurons, REMS essentially does not appear [26], [29], [95].

Rempe et al. [44] considered both circadian and homeostatic drives in their model. Different than our model, the circadian drive in their model directly inhibited neurons in sleep promoting areas (VLPO in their model) as opposed to through ORX-ergic neurons as we did. Both models support the prediction that sleep-wake transitions are likely to be controlled by the sleep active populations in conjunction with the homeostatic and circadian inputs. As in the model by Diniz Behn et al. [40], Rempe et al. [44] also showed that loss of ORX-ergic inputs lead to narcoleptic like behavior. Further they described the phase relationship needed between the circadian drive and the onset of sleep for the occurrence of sleep-onset REMS (SOREM). They showed that when circadian input was low, noise to the system may initiate SOREM by switching the control of the wake to sleep transition from the sleep promoting neurons (VLPO in their model, POAH in ours) to the wake promoting neurons (AMIN in their model, MRF in ours). With the standard parameters, our model does not completely support the conclusion by Rempe et al. as the REM-off neurons in our model would continue to provide inhibition to REM-on neurons preventing SOREM. Our model suggests, instead, that SOREM activity may be related to premature input from GABA-SNr. The SOREM activity as reported by Rempe et al. could be observed in our model by either changing the parameters associated with REM-off neurons to make them excitable, but silent in the absence of input, or by including an inhibitory synaptic input on them from CRF (similar to the eVLPO to REM-off inhibitory synapse in the Rempe et al. model); this extrapolation may be supported by our other experimental findings as reported in the results and discussed in different sections.

Our model agrees with the Rempe et al. finding that REM-off neurons are silent in REMS due to the reciprocally inhibitory connections between REM-off and REM-on neuron groups. The difference between our models lies in how REM-off neurons behave during wake, what triggers them to become silent and what triggers REM-on neurons to become active. In the Rempe model, REM-off neurons were silent during wake and become activated at sleep due to removal of inhibition from e-VLPO. Then the transitions between REMS and NREMS in their model were governed by the intrinsic dynamics of the REM-off population and the gradual re-introduction of inhibition from e-VLPO. Our model makes a different suggestion that the transition to REMS is not governed by the REM-off population but is instead controlled by blocking its inhibition to REM-on neurons. So while both models have a similar output, the sequence of events and causes of the transitions are suggested to be for different reasons.

Using a firing rate model, Phillips and Robinson [39], [41] investigated many aspects of sleep-wake dynamics. In particular, they used a flip-flop model of sleep and wake promoting areas to study the effects of sleep deprivation and the impacts of different types of external stimuli. Our study agrees with some of their findings regarding sleep deprivation. In particular, they also found that depending on the phase of the circadian rhythm at which sleep recovery began following sleep deprivation, brief awakenings could occur. While including a homeostatic and circadian drive, their model did not investigate REMS activity. Diniz Behn and Booth [43] investigated how simulation of micro-injection of specific neurotransmitter agonists or antagonists affected the quantitative properties of sleep-wake cycles such as the frequency or duration of REMS bouts, the duration of the wake state and the percentage of time spent in NREMS. Their model did not consider in depth the role of ORX-ergic input or the circadian rhythm. The study by Postnova [42] considered an excitatory loop consisting of ORX-ergic and glutamate-ergic neurons to investigate the potential roles of synaptic depressions on the ORX-ergic synapse. Their findings add to the body of evidence suggesting that ORX-ergic activity is critical to the stability of sleep-wake cycles. Fleschner et al. [45] recently proposed a few different models of the circadian drive that account for the observed behavior of both nocturnal and SCN-lesioned rats. Their models highlight the importance of feedback projections from the wake-sleep circuit back to the SCN in order to modulate the circadian drive.

Although many of the models, including ours, apparently differ from one another, they do serve a complementary purpose in helping to understand the underlying neuronal mechanisms for the regulation of wake-NREMS-REMS. These differences are obvious either in the choice of intrinsic characteristics ascribed to a particular neuronal group, the choice of which neuronal groups to include and the choice of synaptic interactions between these groups. Our model relies maximally on actual cellular data obtained *in vivo*, a significant proportion from our group, and considers the basic brainstem reticular sleep and waking neuronal circuitry which was not fully accounted for in earlier models. An important difference between our model and that of others is that we consciously chose to model the brainstem reticular wake (MRF in our model) and sleep (CRF in our model) active neurons, which are fundamental for generating sleep-waking [1] and their inputs on the REM-on and REM-off neurons, whose interaction forms the basis for REMS regulation. Different than other models, our model explores how ORX neurons balance circadian input [73], inhibition from POAH [67] and feedback inhibition from REM-off neurons [96]. As a result, our model highlights the variety of ways in which modulation of inputs to or from ORX-ergic neurons can destabilize normal sleep-wake cycling. For example, our model shows how modulation of the strength of the ORX-ergic inputs to the POAH neurons or changes in the strength of the homeostatic drive play a significant role in brief awakenings, shifting the onset (circadian) phase and changing the ratio of wake to sleep (Fig. 10 and Fig. 11). Another difference of our model from other models is in how we chose to model REM-off neurons as being intrinsically active and how they become silent during REMS. This assumption has the consequence that the transition to sleep never results in SOREM activity and it also more easily reveals the potential role of pre-synaptic inhibition from the GABA-SNr in initiating REM-on activity.

Finally, as discussed above, our model helps to explain the mechanism and the causality of the simultaneous cessation of REM-off neurons and activation of REM-on neurons for REMS regulation. Thus, it can be used to make several experimentally testable predictions involving the roles of specific synaptic inputs within the wake-NREMS-REMS neuronal circuit. This might help explain the existence of several neuronal connections and their activity during normal and altered states. Additionally, it suggests that the mechanisms that control the transitions to wake, NREMS and REMS depend on a very delicate balance between the circadian input and the homeostatic drives that are mediated directly and indirectly through the activity of several areas of the brain including ORX-ergic neurons, which has been at least tested by us. The demonstrated consistency and robustness of our model output with empirical data suggests that this model may serve as a candidate starting point for further investigation of neuronal mechanisms of regulation of waking-NREMS-REMS during normal as well as during patho-physiological states.

## Supporting Information

Appendix S1 Equations and parameters used for simulations. This file contains the model equations that were used. Simulations were conducted using XPP.
(DOC)
