# Supplementary material for: A Mathematical Model towards Understanding the Mechanism of Neuronal Regulation of Wake-NREMS-REMS States
Source: PLoS One. 2012 Aug 8;7(8):e42059. doi: 10.1371/journal.pone.0042059 (PMC3414531; doi:10.1371/journal.pone.0042059)
Supplement: Appendix S1 — Equations and parameters used for simulations. This file contains the model equations that were used. Simulations were conducted using XPP. (DOC) [file pone.0042059.s001.doc]

**Appendix:**

**Model Equations:**

The Morris-Lecar equations were used to describe the dynamics of individual neuron groups. These equations, with parameters given below, produce oscillations in which *v* varies between -1 and 1. For the figures, we have rescaled the output to vary between 0 and 1.

$$v_{i}^{'}=I_{app}+g_{l}(v_{l}-v)+g_{k}(v_{k}-v)+g_{ca}m_{\infty}(v)(v_{ca}-v)$$

$$w_{i}^{'}=\lambda_{\infty}(v)(w_{\infty}(v)-w)$$

where,

$$m_{\infty}(v)=0.5\left[1+\tanh\left(\frac{v-v_{1}}{v_{2}}\right)\right]$$

$$\lambda_{\infty}(v)=\varphi\cosh\left(\frac{v-v_{3}}{2v_{4}}\right)$$

$$w_{\infty}(v)=0.5\left[1+\tanh\left(\frac{v-v_{3}}{v_{4}}\right)\right]$$

The following common parameter values were used for all groups POAH, MRF, CRF, ORX, R-On, R-off and GABA-LC: v_l_=-0.1, v_k_=-0.7, v_ca_=1, v_1_ = -0.01, v_2_=0.15, v_4_=0.145,g_l_=0.5, g_k_=2, gc_a_=1.33. The value v_3_ is -0.15 for POAH, CRF and GABA-LC, 0 for R-on, 0.1 for MRF and ORX and 0.12 for R-on. The value φ= 0.01 for POAH, 0.05 for CRF, 0.1 for R-on, R-off and GABA-LC and 1 for MRF and ORX. The value I_app_=-0.1 for GABA-LC, 0 for POAH, MRF, R-on, R-off and ORX and 0.7 for CRF.

**Synaptic Equations:**

$$I_{syn}=g_{syn}s_{\infty}\left(v_{pre}\right)\left[v_{post}-E_{syn}\right]$$

$$s_{\infty}(v)=0.5\left[1+\tanh\left(\frac{v-v_{5}}{v_{6}}\right)\right]$$

Here v_pre_ refers to the pre-synaptic group and v_post_ the post-synaptic group. For inhibitory synapses Esyn=-0.7; for excitatory synapses Esyn =0 for ORX to R-off and MRF and CRF to POAH, 0.15 for R-on to GABA-LC, 0.2 for CRF to R-on, and 0.4 for MRF to R-off. The value v_5_= -0.1 for MRF, CRF, R-off and ORX, 0 for POAH and GABA-LC and 0.1 for R-on. The value v_6_= 0.2 for all synapses. The synaptic strength from MRF are 0.5 , 1, 0.5 and 0.5 to POAH, CRF, R-on and R-off; from CRF are 0.5, 0.2 to POAH and MRF. When the REM homeostatic input is present for Fig. 9, the CRF to R-on strength is 0.57 and 0 otherwise; from POAH are 1 and 0.5 to ORX and MRF; from ORX are 1 to POAH and MRF and 0.5 to R-off; from R-off are 0.5 to R-on and 1 to ORX for Fig. 13; from R-on to GABA-LC is 1; and from GABA-LC to R-off =0.5.

**Circadian and Homeostatic Input:**

The sinusoidal oscillating function for circadian input to ORX is chosen according to [62] as

$$C(t)=0.97\sin(\omega t)+0.22\sin(2\omega t)+0.07\sin(3\omega t)+0.03\sin(4\omega t)+0.01\sin(5\omega t)$$

$\omega=\frac{2\pi}{24}$The parameter g_cir_=0.3 unless otherwise noted. The parameters associated with the homeostatic drive to POAH are τ_1_= 20,000, τ_2_=1800 and g_h_=5. The parameters for the REM homeostat used for Fig. 9 are τ_3_=1, τ_4_=10, τ_5_=700, τ_6_=3000.
